# Assessment of early kidney injury caused by asymptomatic bacteriuria in children with type 1 diabetes

**DOI:** 10.1186/s12887-022-03689-1

**Published:** 2022-11-07

**Authors:** Gihan M. Bebars, Asmaa N. Mostafa, Hend M. Moness, Reem A. Abdel Aziz

**Affiliations:** 1 Department of Pediatrics, Faculty of Medicine, Minia University, Minia, 61111 Egypt; 2 Department of Clinical Pathology, Faculty of Medicine, Minia University, Minia, Egypt

**Keywords:** Asymptomatic bacteriuria, NGAL, Hs-CRP, GFR

## Abstract

**Introduction:**

Infection is one of the most frequent causes of morbidity and mortality in diabetic patients. Some microorganisms become more virulent in a high glucose concentration. Diabetics are more likely to have asymptomatic and symptomatic bacteriuria. NGAL is secreted in high concentrations into the blood and urine within two hours of AKI.

**Objectives:**

The aim of the study is early detection of UTI in type1diabetic children through screening of their urine samples, and measurement of NGAL urinary levels in cases with asymptomatic bacteriuria for early detection of AKI to prevent serious complications.

**Patients and methods:**

One thousand twenty-two known diabetic children on regular follow up in endocrine outpatient clinic at Minia Children University hospital were screened for UTI. From them only 52 diabetic children were diagnosed as asymptomatic bacteriuria (group I), 52 diabetic children with normal urine analysis (group II) and 52 apparently healthy children, age and sex matched, served as controls (group III). CBC, Renal function test, HbA1c, hs- CRP, Albumin/creatinine ratio, urine examination, urine culture, GFR and urinary NGAL were done to all children.

**Results:**

Thirty-seven females (71.2%) had asymptomatic bacteriuria, Hs CRP and urinary NGAL were significantly higher, while GFR was significantly lower in diabetic children with bacteriuria than the other two groups. For diabetic children with bacteriuria, (AUC) for NGAL was 1 with optimal cutoff value of > 44.1 (Sensitivity 100% and Specificity 100%) while AUC for hsCRP was 0.887 with optimal cutoff value of > 1 (Sensitivity 82.69% and Specificity 90.38%).

**Conclusion:**

Routine urine analysis should be done for all diabetic children even if they are asymptomatic. NGAL and hsCRP are non-invasive methods that could detect early renal injury in these patients thus, early, and proper management of UTI should be started to prevent renal injury.

## What is known?

- Diabetics are more likely to have asymptomatic and symptomatic bacteriuria.
- Neutrophil gelatinase associated lipocalin (NGAL)has been identified as one of the earliest and the most indicative biomarkers of acute kidney injury (AKI).

## What is new?

- We measured NGAL to detect both urinary tract infection (UTI) and AKI in diabetic children with asymptomatic bacteriuria (ASB).
- We studied the correlation between ASB and the degree of glycemic control, and NGAL.

## Introduction

Patients with type 1 diabetes, even with proper management and glycemic control, may develop a variety of diabetic sequel [1]. One of these sequels is infection which increases the frequency of morbidity and mortality among diabetic children, and urinary tract infection (UTI) is considered as one of most common infections encountered in them [2]. Moreover, Diabetics are more likely to have asymptomatic and symptomatic bacteriuria (ASB) [1].

Elevated serum and urine glucose levels that impedes neutrophil diapedesis and phagocytosis, as well as defective host immune factors. Furthermore, the urinary retention of diabetic neuropathy, nephropathy, vesicourethral reflux and renal papillary necrosis are additional factors in the development of diabetic UTIs [3].

Asymptomatic bacteriuria (ASB) is defined as the presence of $\ge$ 10^5^ colony-forming units/ml of one or more species of bacteria in a culture of clean-voided midstream urine obtained from a patient without symptoms of urinary tract infection [4].

The prevalence of ASB in diabetic patients is four times higher than the general population [5].

Diabetic patients are at higher risk of suffering from complications as bacteremia, pyelonephritis, renal abscess, renal papillary necrosis, severe kidney damage or renal failure. So, early diagnosis and correct treatment of UTI in diabetic patients is very essential [6].

One of the most indicative biomarkers for acute kidney injury (AKI) is Neutrophil Gelatinase Associated Lipocalin (NGAL), also called lipocalin-2, produced from the nephron in response to tubular epithelial damage [7]. It is involved in the sequestration of iron blocking bacterial growth. It is expressed in neutrophils, kidney, prostate, respiratory and gastrointestinal epithelia. It is easily excreted and detected in urine because it is protease resistant. It is secreted in high concentrations into the blood and urine within two hours of AKI. Moreover, it is easily excreted and detected in the urine as it is protease resistant [8].

The aim of the study is early detection of UTI in type1diabetic children in Minia governorate, through screening of their urine samples, measurement of high sensitive C reactive protein (hsCRP) and NGAL urinary levels (as it has a great significance in AKI) in cases with detected asymptomatic bacteriuria for early detection of AKI to prevent serious complications among this group of children and improving their quality of health.

## Methods

### Patients

This is a prospective study, conducted on 1022 children with type I diabetes mellitus who were on regular follow up in endocrine outpatient clinic at Minia Children University hospital. From them only 52 children were diagnosed as asymptomatic bacteriuria (group I) (Bacteriuria is assumed if a single bacterial species is isolated in a concentration greater than 100,000 colony forming units (CFU) per milliliter of urine in clean-catch midstream urine specimens) [4]. Their age ranged from 3 to 18 years and were on regular insulin therapy. Only diabetic children with normal albumin / creatinine ratio were included in our study.

Another 52 diabetic children without bacteriuria, age and sex matched and they were also on regular insulin therapy and had normal albumin / creatinine ratio (group II) 52 apparently healthy children, age and sex matched, served as a control group (group III). Our patients were collected from the endocrine outpatient clinic and from the inpatient endocrinology unit of Minia Children University Hospital. Controls were collected from outpatient general clinic of the same hospital over the period from January 2021 to November 2021. A total 104 children were divided into 3 groups:

#### Group I

Included 52 children with type 1 diabetes according to Standard ADA criteria 2010 and had regular follow up in Pediatric Endocrinology outpatient's clinic of Minia University Children Hospital [9]. All of them had asymptomatic bacteriuria with positive urine cultures.

#### Group II

Included 52 children with type 1 diabetes according to Standard ADA criteria 2010 and had regular follow up in Pediatric Endocrinology outpatient's clinic of Minia University Children Hospital [9]. All of them had normal urine analysis with negative urine cultures.

#### Group III

Included 52 normal children who were confirmed to be non-diabetic by measuring their fasting blood glucose according to ADA criteria 2010 [9]. They all had normal urine analysis.

We have excluded from the study; diabetic children below 3 years of age, children with symptoms of UTI at the time of the study (burning micturition, frequency, urgency and change of color, volume, or odor of urine), patients with history of urologic disease (stricture urethra, posterior urethral valve, and meatal stenosis) children with other autoimmune disease and those with other chronic diseases and children having micro or macro- albuminuria were excluded from the study.

All studied children were subjected to history taking including demographic data, diabetes focused history including; age of onset, duration, presentation of diabetes, insulin therapy (regarding type of insulin, dose and frequency), history suggestive of urinary tract infection (burning micturition, change of urine color or odor, frequency or urgency), history suggestive of chronic diabetic complication like ocular, cardiovascular system (CVS), Peripheral neuropathy manifestations, and history of other medications i.e. antibiotics or any other associated disease. Clinical examination was done to all participating children including anthropometric measurements, general and systemic examination. Finally, laboratory investigation divided into (a) routine investigations: complete blood count (CBC), Renal function test, HbA1c, hs- CRP, albumin/creatinine ratio in urine and urine analysis (b) special investigations: urine culture, glomerular filtration rate (GFR) and measuring of urinary NGAL by enzyme-linked immunosorbent assay (EIA).

### Sampling protocol

Six ml of venous blood were withdrawn from every child participating in the study under complete aseptic condition.

- ➢ 2 ml blood were collected on sterile vacutainers containing EDTA solutions tube for CBC assay.
- ➢ 2 ml blood into sterile vacutainers containing EDTA solutions tube for HbA1c assessment.
- ➢ 2 ml blood were put into serum separator gel tube, sample was allowed to clot for 30 min at 37 °C before centrifugation for 15 min at 3,500 rpm. The expressed serum was used for measurement of renal function test and hs-CRP.

Clean midstream urine specimens were collected for all children.

### Methodology

A) Routine investigations:
  ➢ Complete blood count (CBC): It was determined by automated cell counter (Celltac ES, Nihon Kohden Corporation, Automated hematology analyzer, Japan).
  ➢ Renal function test and albumin/ creatinine ratio were assayed using fully automated clinical chemistry auto-analyzer system (Auto-analyzer Selectra proM, ELITech Group, clinical chemistry automation systems, Finland). Albumin/creatinine ratio may be
    ➢ Normal level up to 0.3 mg/dl.
    ➢ 0.3 – 3 mg/dl microalbuminuria.
     ➢ > 3 mg/dl macroalbuminuria
  ➢ Hs-CRP and HbA1c were measured using Nephelometry (GENIUS PA54—Specific Protein Analyzer, Chain).
B) Special investigation:
  ➢ Urine culture was done on both MacConkey media (TITAN Biotech LTD, India). Each urine specimen was cultivated on both media by 1 µl calibrated loop, to estimate the approximate number of bacteria in each specimen, both plates were incubated at 37ºC for 24 h and then examined for the growing organisms and colony count was done. if no growth the plate was left for 48 h before discarding it.
  ➢ Urinary NAGL by EIA (kit was supplied by bioassay technology laboratory cat. No E1719Hu).

### Estimated Glomerular Filtration Rate (eGFR)

Was calculated by the equation: eGFR = k x (height in cm) ÷ serum Cr.

$$\mathrm{k }= 0.55$$

### Statistical methods

Data were analyzed using the statistical package for the Statistical Package for Social Sciences (SPSS) version 25. Quantitative variables were summarized in the form of mean ± standard deviation (SD) if normally distributed. On the other hand, qualitative variables were summarized in the form of frequencies and percentages. The independent samples t-test was used to compare quantitative data [10], while the Chi-square or Exact test was used to compare categorical data [11]. Pearson’s or Spearman’s correlation coefficient was used to analyze the correlation between variables. ROC curve analysis was done to calculate the area under the curve, optimal cutoff point, sensitivity, specificity, positive predictive value, negative predictive value, and accuracy of variables predicting cases. *P* values ≤ 0.05 were considered statistically significant.

### Ethical considerations

The study had the approval of the Minia College of Medicine Ethical Committee. All the actions performed were according to the Helsinki Declaration and its modifications. Before patients' enrollment in the study, written informed approval was obtained from their parents.

## Results

Concerning the demographic data, diabetic group showed predominance of female sex (71.2%) Duration of diabetes was 1-8 (4.5±1.8) in diabetic children with bacteriuria (Group I) and 1-8 (4.2±1.7) in diabetic children without bacteriuria (Group II) (Table 1). Glucosuria was detected in 10 (19.2%) and 7 (13.5%) in both groups I and II respectively. Pyuria was seen in 25 (48.1%) (group I), with higher frequency in females (80%). (Table 2). All the collected urine samples were culture positive (group I). *E. Coli* was the most frequently isolated organism (22) from urine cultures (42.31%), 15 cultures revealed *Kelebsiella pneumonie* (28.85%), 8 with *Staph. Saprophyticus* (15.38*%*)*,* and 7 cultures detected *Enterobacter* (13.46%). Regarding diabetes control, serum levels of HbA1c were 7.8±0.8 and 7.6±0.9 in both diabetic groups respectively. CRP was positive in 6 (11.5%) diabetic children with bacteriuria. Both hs CRP and NGAL levels were significantly higher in diabetic children with bacteriuria (group I) than the other two groups (*p*<0.001), while GFR was significantly lower in diabetic children with bacteriuria (*p*<0.001) (Table 3).

**Table 1:** Demographic and clinical data of the studied groups

		**DM with bacteriuria**	**DM without bacteriuria**	**Control**	***P* value**
		***N* = 52**	***N* = 52**	***N* = 52**	
**Age (years)**	*Range* *Mean* ± *SD*	(7–14) 11.5 ± 2.2	(7–14) 11.5 ± 2.1	(7–14) 11.6 ± 2.3	*0.989*
**Sex**	*Male* *Female*	15(28.8%) 37(71.2%)	16(30.8%) 37(69.2%)	17(32.7%) 35(67.3%)	*0.911*
**Weight**	*Range* *Mean* ± *SD*	(21–36) 27.3 ± 4.2	(21–36) 28.8 ± 4.5	(22–36) 28 ± 4.3	*0.210*
**Height**	*Range* *Mean* ± *SD*	(116–164) 139.7 ± 13.8	(120–162) 141.8 ± 14.5	(116–164) 141.1 ± 14.6	*0.746*
**BMI**	*Range* *Mean* ± *SD*	(12.8.2–15.6) 14 ± 0.8	(13.3–16) 14.3 ± 0.8	(13–16.4) 14.1 ± 1	*0.144*
**Duration of DM (years)**	*Range* *Mean* ± *SD* *Median (IQR)*	(1–8) 4.5 ± 1.8 4.5/ (3–6)	(1–8) 4.2 ± 1.7 4/ (3–4.5)	––-	*0.325*

Independent Samples T test for quantitative data between the two groups

Chi square test for qualitative data between the two groups

*BMI* Body mass index, *DM* Diabetes mellitus

^*^ Significant level at *P* value < 0.05

**Table 2:** Urine analysis data of the studied groups

		**DM with bacteriuria**	**DM without bacteriuria**	**Control**	***P* value**
		***N* = 52**	***N* = 52**	***N* = 52**	
**Pus cells**	*-Ve* + *Ve*	27(51.9%) ^**a**^ 25(48.1%)	52(100%) ^**b**^ 0(0%)	52(100%) ^**b**^ 0(0%)	< 0.001*
**Pus cells number**	*Range* *Mean* ± *SD* *Median (IQR)*	(5–90) 37.6 ± 30 25/ (10–70)	––	––	*––*
**Glucosuria**	*-Ve* + *Ve*	42(80.8%) ^**a**^ 10(19.2%)	45(86.5%) ^**a**^ 7(13.5%)	52(100%) ^**b**^ 0(0%)	0.005*

Fisher’s exact test for qualitative data between the two groups

Superscript with different small letters refer two significance difference between the two groups

^*^ Significant level at *P* value < 0.05

**Table 3:** Laboratory data of the studied groups

		**DM with bacteriuria**	**DM without bacteriuria**	**Control**	***P* value**
		***N* = 52**	***N* = 52**	***N* = 52**	
**HbA1c**	*Range* *Mean* ± *SD*	(6.7–10) ^**a**^ 7.8 ± 0.8	(6–9) ^**a**^ 7.6 ± 0.9	(4.6–6.5) ^**c**^ 5.5 ± 0.5	< 0.001*
**TLC**	*Range* *Mean* ± *SD*	(4.5–9.8) 7 ± 1.6	(5–9) 7.1 ± 1.3	(5.5–8.5) 7.2 ± 0.9	*0.855*
**Urea**	*Range* *Mean* ± *SD*	(13.5–40.7) 28.5 ± 7.8	(15.2–39.3) 27.4 ± 7.4	(15.1–39.8) 27.8 ± 7.1	*0.744*
**Creatinine**	*Range* *Mean* ± *SD*	(0.57–0.99) 0.74 ± 0.11	(0.5–1) 0.7 ± 0.1	(0.51–0.96) 0.71 ± 0.14	*0.394*
**NGAL**	*Range* *Mean* ± *SD*	(46–92) ^**a**^ 60.9 ± 12.2	(17–45) ^**b**^ 31.5 ± 6.6	(15.5–44.1) ^**b**^ 28.2 ± 8.9	< 0.001*
**hs-CRP**	*Range* *Mean* ± *SD*	(0.8–3) ^**a**^ 1.5 ± 0.6	(0.8–1.6) ^**b**^ 1 ± 0.2	(0.8–1.1) ^**b**^ 0.9 ± 0.1	< 0.001*
**GFR**	*Range* *Mean* ± *SD*	(70–95) ^**a**^ 83 ± 6.8	(115.7–134.8) ^**b**^ 125.1 ± 5.7	(115–135) ^**b**^ 125 ± 6.1	< 0.001*
**CRP**	*-Ve* + *Ve*	46(88.5%) ^**a**^ 6(11.5%)	52(100%) ^**b**^ 0(0%)	52(100%) ^**b**^ 0(0%)	0.002*

*TLC* Total leucocytic count, *NGAL* Neutrophil Gelatinase Associated Lipocalin, *hs-CRP* High sensitive c reactive protein, *GFR* Glomerular filtration rate

Independent Samples T test for quantitative data between the two groups

Fisher’s exact test for qualitative data between the two groups

Superscript with different small letters refer two significance difference between the two groups

^*^ Significant level at *P* value < 0.05

**Fig. 1:**
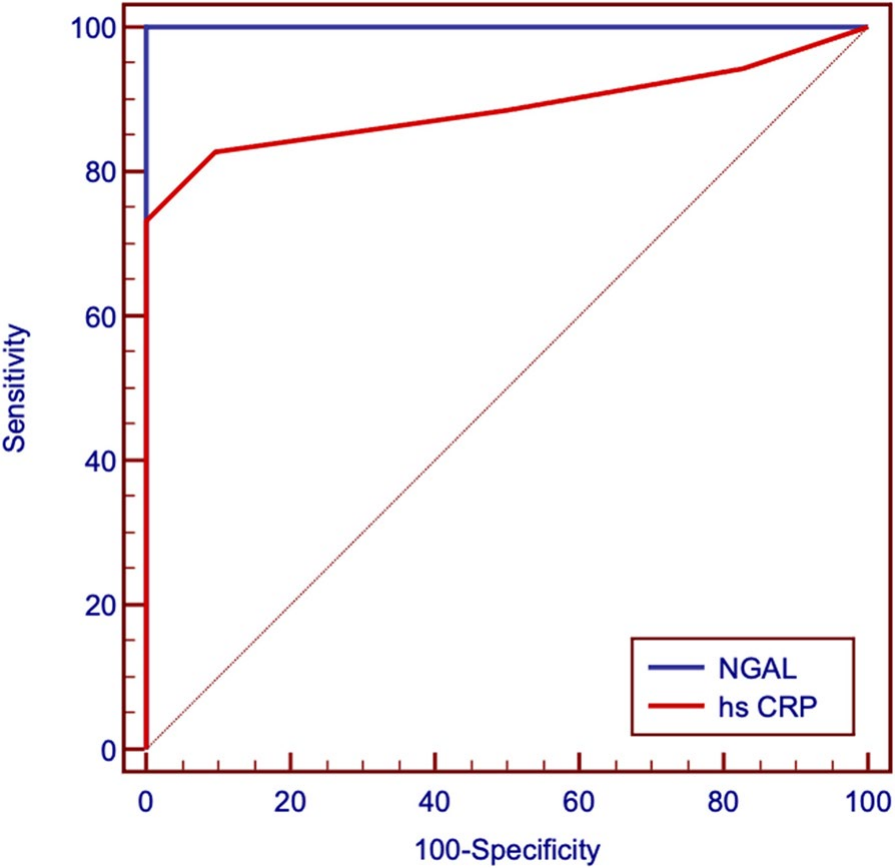
ROC curve analysis for prediction of diabetic cases with bacteriuria

ROC curve analysis for prediction of cases revealed that the area under the curve (AUC) for NGAL was 1 with optimal cutoff value of > 44.1 (Sensitivity 100% and Specificity 100%) while AUC for hsCRP was 0.887 with optimal cutoff value of > 1 (Sensitivity 82.69% and Specificity 90.38) (Table 4 and Fig. 1). In diabetic children with bacteriuria, NGAL was positively correlated with hsCRP. Both NGAL and hsCRP were positively correlated with HbA1c, number of pus cells, and serum creatinine. Both NGAL and hsCRP were negatively correlated with GFR. GFR was negatively correlated with HbA1c and number of pus cells, urea, and creatinine (Table 5). In diabetic patients without bacteriuria, only hsCRP was positively correlated with HbA1c and the duration of diabetes (Table 6).

**Table 4:** ROC curve analysis for prediction of diabetic cases with bacteriuria

	**NGAL**	**hs CRP**
Optimal cutoff	> 44.1	> 1
AUC	1	0.887
**95% CI**	0.965–1	0.810–0.941
**P value**	< 0.001*	< 0.001*
**Sensitivity**	100	82.69
**Specificity**	100	90.38
**PPV**	100	89.6
**NPV**	100	83.9
**Accuracy**	100	86.5

*AUC* Area Under Curve, *CI* Confidence Interval, *PPV* Positive Predictive Value, *NPV* Negative Predictive Value

^*^ Significant level at *P* value < 0.05

**Table 5:** Correlation coefficient between the laboratory data in diabetic patients with bacteriuria

DM with bacteriuria	NGAL		hs CRP		GFR	
	**r**	***P* value**	**r**	***P* value**	**r**	***P* value**
**hs CRP **^**(P)**^	***0.992***	< 0.001*				
**GFR **^**(P)**^	***-0.895***	< 0.001*	***-0.905***	< 0.001*		
**Duration **^**(P)**^	*-0.204*	*0.146*	*-0.210*	*0.134*	*0.148*	*0.294*
**HbA1c **^**(P)**^	***0.978***	< 0.001*	***0.980***	< 0.001*	***-0.923***	< 0.001*
**pus cells number **^**(P)**^	***0.958***	< 0.001*	***0.942***	< 0.001*	***-0.765***	< 0.001*
**Presence of pus cells **^**(S)**^	***0.778***	< 0.001*	***0.785***	< 0.001*	***-0.783***	< 0.001*
**Creatinine **^**(P)**^	**0.757**	< 0.001*	**0.781**	< 0.001*	**-0.779**	< 0.001*
**Urea **^**(P)**^	**0.433**	0.001*	**0.426**	0.002*	**-0.496**	< 0.001*

*(P)* Pearson’s correlation, *GFR* glomerular filtration rate, *(S)* Spearman’s correlation

^*^: Significant level at *P* value < 0.05

**Table 6:** Correlation coefficient between the laboratory data in diabetic patients without bacteriuria

DM without bacteriuria	NGAL		hs CRP		GFR	
	**r**	***P* value**	**r**	***P* value**	**r**	***P* value**
**hs CRP**	*0.187*	*0.185*				
**GFR**	*-0.125*	*0.378*	*0.107*	*0.449*		
**duration**	*0.225*	*0.108*	***0.299***	0.031*	*-0.031*	*0.828*
**HbA1c**	*0.127*	*0.370*	***0.671***	< 0.001*	*0.146*	*0.300*
**Urea**	*0.157*	*0.267*	*0.171*	*0.226*	*0.109*	*0.443*
**Cr**	*-0.027*	*0.848*	*-0.036*	*0.802*	*0.168*	*0.232*

Pearson’s correlation

^*^: Significant level at *P* value < 0.05

## Discussion

Urinary tract infections are one of the leading causes of acute kidney injury. UTI can be either symptomatic or asymptomatic. Symptoms could range from mild irritative voiding to bacteremia, sepsis, shock or even death [7].

NGAL is an excellent biomarker for early prediction, monitoring and prognosis of AKI. It is expressed in nephrons. It also appears to play an important role in the repair and regeneration of kidney tubule cells after AKI [7].

Our study included 52 diabetic children with asymptomatic bacteriuria; all these children had greater than 100,000 colony forming units (CFU) per milliliter of urine in clean midstream urine specimens, with higher frequency within female children. E. Coli was the most frequently isolated organism from urine cultures (42.3%), Klebsiella pneumoniae (28.85%), Staph. Saprophyticus (15.38*%*), and Enterobacter (13.46%).

Various studies confirmed the role of the female sex as a risk factor for ASB in diabetics. The study by Turan et al. noticed that 77.2% of females were positive for ASB [12]. Also, results from the studies by Jha et al. and Alebiosu et al. reported similar results (70% and 72.7% respectively), while Banerjee et al., reported a lower frequency (59%) [13–15].

Abdolrahim et al.2018, concluded that ASB and pyuria were more prevalent among diabetic children as 10.7% of participants had positive urine culture, indicating UTI with a higher frequency among females than males. So, they recommended regular screening for pyuria and asymptomatic bacteriuria in diabetic children to help diagnose and prevent complications from urinary tract infections [1].

Another Egyptian comparative study stated that the prevalence of ASB was higher among diabetics than controls (30% versus 14%, *p* < 0.01) and it was also higher in female patients (*p* < 0.001). The most common isolates in urine culture were *E. coli* in patients (30%) and Pseudomonas in controls (57.1%). Gram positive isolates were detected in 46.7% of diabetic patients but not in controls [16].

Moreover, a study conducted in Sudan showed the growth of E. coli (56.4%), K. Pneumoniae (23.0%), and E. faecalis (12.8%) in the urine culture of diabetic children [17].

Our studied children, HbA1c serum levels were 7.8±0.8 and 7.6±0.9 in diabetic children with and without bacteriuria respectively. Both Hs CRP and urinary NGAL were significantly higher in diabetic children with bacteriuria, these results were also associated with significantly decreased GFR. (*p*<0.001).

ROC curve analysis for prediction of cases revealed that the area under the curve (AUC) for NGAL was 1 with optimal cutoff value of > 44.1 (Sensitivity 100% and Specificity 100%) while AUC for hsCRP was 0.887 with optimal cutoff value of > 1 (Sensitivity 82.69% and Specificity 90.38).

In diabetic children with bacteriuria, NGAL and hsCRP were positively correlated with HbA1c, number of pus cells, urea, and creatinine, but negatively correlated with GFR and duration of diabetes. GFR was negatively correlated with HbA1c, pus cells, urea, and creatinine.

Marzuillo et al., concluded elevated NGAL levels in type 1 diabetes mellitus presented with DKA [18].

Horváth et al., concluded that NGAL had a leading role in diagnosing and differentiating UTIs based on 131 observational, comparative trials on pediatric population [19].

In accordance with our findings, Ichino et al. observed increased concentration of renal NGAL mRNA and protein levels also, Yilmaz et al. demonstrated higher NGAL levels in UTI group than the control group [20, 21].

Another recent study found that urinary NGAL excretion is higher in type 1 diabetic patients than controls [22].

Results of Munilakshmi et al., revealed that NGAL and hs-CRP levels were higher in diabetic patients with positive correlations with other markers such as serum creatinine, GFR, and hs-CRP. and they concluded that the occurrence of UTI in diabetes is related to poor glycemic control which may promote the growth of pathogenic bacteria. The increased serum NGAL could be an important sensitive and direct biomarker for detecting acute pyelonephritis and can be used for monitoring the treatment response of diabetes with AKI patients [7].

Other studies were not in consistence with our study as they found no association between UTI and glycemic control, but this may be due to the variety of study populations and the selection criteria used in these studies [16, 23–28]

We do have some limitations in our study, like it is a single centered study and needs to include a larger number of children at different age groups. However, despite these limitations, we can recommend measurement of urinary NGAL and hsCRP serum levels for early detection and follow up of renal parenchymal involvement in asymptomatic UTIs.

## Conclusion

Routine urine analysis should be done for all diabetic children even if they are asymptomatic to detect cases with ASB. Urinary NGAL and hsCRP are non-invasive methods that can detect early renal injury from ASB in diabetic children so, early, and proper management should be started without delay to prevent progressive renal damage.

### Impact on society

Urinary NGAL and serum hsCRP can detect early renal injury from ASB in diabetic children so, early, and proper management should be started without delay to prevent progressive renal damage.

## Data Availability

The datasets generated and/or analyzed during this study are not publicly available as some of these data will be used for another research study. However, the datasets can be shared from the corresponding author on reasonable request.

## Abbreviations

ADA: American Diabetes Association
AKI: Acute kidney injury
ASB: Asymptomatic bacteriuria
AUC: Area under the curve
BMI: Body mass index
CBC: Complete Blood Count
CFU: Colony forming units
CI: Confidence Interval
CVS: Cardiovascular system
DM: Diabetes mellitus
e-GFR: Estimated glomerular filtration rate
EDTA: Ethylene Diamine Tetra acetic Acid
EIA: Enzyme-linked immunosorbent assay
GFR: Glomerular filtration rate
Hs CRP: High C reactive protein
NGAL: Neutrophil Gelatinase Associated Lipocalin
NPV: Negative Predictive Value
PPV: Positive Predictive Value
ROC curve: Receiver operating characteristic curve
SD: Standard deviation
TLC: Total leucocytic count
UTI: Urinary tract infection
